# Supplementary material for: A Likelihood Approach for Real-Time Calibration of Stochastic Compartmental Epidemic Models
Source: PLoS Comput Biol. 2017 Jan 17;13(1):e1005257. doi: 10.1371/journal.pcbi.1005257 (PMC5240920; doi:10.1371/journal.pcbi.1005257)
Supplement: S1 File — (TAR.GZ) [file pcbi.1005257.s014.tar.gz › HSPH_Online-SI-Revision/Additions/EnKF08/n100-extreme_noisy/n100-extreme_noisy_EnKF2_table0.pdf]

| | | | | | | | | | | | | | | | | | | | | | | | | | | | | | | |
|---|---|---|---|---|---|---|---|---|---|---|---|---|---|---|---|---|---|---|---|---|---|---|---|---|---|---|---|---|---|---|
| 1 | newly 3 weeks spec | 7368.61 | 1695.94 | {0., 2503.} | 93.8651 | 2.36223 | newly 3 weeks cumul | 27 409.9 | 15 167.5 | {5057., 29 300.} | 78.7615 | 0.541663 | newly 5 weeks spec | 4368.49 | 307.06 | {0., 532.} | 94.9967 | 2.61007 | newly 5 weeks cumul | 37 692.1 | 16 193.1 | {5345., 31 188.} | 79.326 | 0.634573 | newly 54 weeks cumul | 51 622.1 | 16 464.5 | {5397., 31 439.} | 80.4407 | 0.743541 |
| 2 | newly 3 weeks spec | 5965. | 1425.67 | {25., 4424.} | 86.435 | 1.26631 | newly 3 weeks cumul | 20 994. | 4635.25 | {373., 12 036.} | 88.3747 | 1.058 | newly 5 weeks spec | 3796. | 1154.96 | {1., 3725.} | 84.4947 | 1.54553 | newly 5 weeks cumul | 29 744. | 7086.05 | {466., 19 042.} | 85.5635 | 1.06948 | newly 50 weeks cumul | 42 279. | 13 450. | {507., 38 396.} | 81.2992 | 1.04278 |
| 3 | newly 3 weeks spec | 6513. | 967.03 | {0., 1913.} | 93.402 | 2.17656 | newly 3 weeks cumul | 27 002. | 11 558.1 | {503., 21 796.} | 83.8875 | 0.936064 | newly 5 weeks spec | 3181. | 389.7 | {0., 543.} | 97.3537 | 3.04353 | newly 5 weeks cumul | 34 839. | 12 533.5 | {540., 26 712.} | 83.7623 | 0.993428 | newly 55 weeks cumul | 41 209. | 13 561.3 | {609., 31 345.} | 84.0561 | 1.02105 |
| 4 | newly 3 weeks spec | 6774. | 925.02 | {9., 1591.} | 93.0886 | 1.74899 | newly 3 weeks cumul | 33 063. | 4691.76 | {211., 5765.} | 91.5953 | 1.37621 | newly 5 weeks spec | 2428. | 399.1 | {0., 1036.} | 95.2092 | 2.17193 | newly 5 weeks cumul | 39 680. | 5693.37 | {227., 8630.} | 91.9678 | 1.41178 | newly 59 weeks cumul | 42 886. | 6522.91 | {236., 12 840.} | 92.1718 | 1.42343 |
| 5 | newly 3 weeks spec | 8431. | 3163.05 | {122., 16 293.} | 93.0586 | 1.20761 | newly 3 weeks cumul | 31 964. | 19 291.1 | {1409., 85 064.} | 97.1174 | 0.823477 | newly 5 weeks spec | 4440. | 981.89 | {3., 5357.} | 92.18 | 1.79722 | newly 5 weeks cumul | 42 439. | 22 010.6 | {1704., 100 165.} | 94.0829 | 0.885705 | newly 56 weeks cumul | 51 326. | 23 373.6 | {1800., 107 909.} | 91.0683 | 0.927116 |
| 6 | newly 3 weeks spec | 7676. | 1646.1 | {273., 3002.} | 84.7223 | 1.12916 | newly 3 weeks cumul | 28 666. | 11 861.2 | {2908., 29 360.} | 74.5752 | 0.641759 | newly 5 weeks spec | 4357. | 433.86 | {38., 830.} | 93.2793 | 1.48976 | newly 5 weeks cumul | 38 952. | 13 110.6 | {2996., 30 469.} | 76.7197 | 0.712207 | newly 54 weeks cumul | 50 195. | 13 700. | {3016., 30 585.} | 78.6145 | 0.786469 |
| 7 | newly 3 weeks spec | 7326. | 860.95 | {30., 1275.} | 95.2633 | 1.63946 | newly 3 weeks cumul | 27 951. | 4857.41 | {442., 7427.} | 93.2947 | 1.27592 | newly 5 weeks spec | 3953. | 339.62 | {0., 372.} | 94.9492 | 2.13561 | newly 5 weeks cumul | 37 123. | 5732.94 | {554., 8506.} | 93.3208 | 1.33805 | newly 52 weeks cumul | 46 099. | 6475.43 | {623., 8932.} | 92.8234 | 1.39307 |
| 8 | newly 3 weeks spec | 7809. | 1152.49 | {0., 1631.} | 93.9161 | 2.13076 | newly 3 weeks cumul | 28 877. | 11 337.1 | {2411., 19 970.} | 76.6467 | 0.707745 | newly 5 weeks spec | 4404. | 353.92 | {0., 524.} | 97.6258 | 2.42337 | newly 5 weeks cumul | 39 237. | 12 355.4 | {2411., 20 848.} | 78.8624 | 0.798207 | newly 54 weeks cumul | 50 550. | 12 724.4 | {2411., 20 998.} | 80.9413 | 0.886821 |
| 9 | newly 3 weeks spec | 8491. | 1069.76 | {0., 1673.} | 92.3974 | 1.90443 | newly 3 weeks cumul | 32 010. | 11 524.1 | {3952., 20 401.} | 78.3844 | 0.705242 | newly 5 weeks spec | 4649. | 295.25 | {0., 405.} | 96.7587 | 2.33024 | newly 5 weeks cumul | 43 099. | 12 389. | {4324., 21 077.} | 81.5119 | 0.794738 | newly 54 weeks cumul | 54 004. | 12 722.2 | {4412., 21 127.} | 83.4564 | 0.872756 |
| 10 | newly 3 weeks spec | 3970. | 2293.88 | {265., 2803.} | 83.594 | 0.68829 | newly 3 weeks cumul | 22 883. | 12 268.3 | {3857., 16 313.} | 76.0914 | 0.503178 | newly 5 weeks spec | 1101. | 800.23 | {7., 1423.} | 71.4814 | 0.83036 | newly 5 weeks cumul | 26 005. | 14 415.3 | {4057., 17 735.} | 75.455 | 0.493361 | newly 58 weeks cumul | 27 241. | 15 839. | {4097., 19 360.} | 72.661 | 0.467838 |
| 11 | newly 3 weeks spec | 6532. | 744.68 | {0., 1408.} | 96.2033 | 2.15209 | newly 3 weeks cumul | 23 257. | 4589.07 | {301., 8137.} | 92.91 | 1.24321 | newly 5 weeks spec | 3974. | 301.3 | {0., 457.} | 97.0533 | 2.59516 | newly 5 weeks cumul | 32 341. | 5360.17 | {310., 9441.} | 92.6259 | 1.32509 | newly 52 weeks cumul | 43 101. | 5954.12 | {310., 9900.} | 93.0937 | 1.40695 |
| 12 | newly 3 weeks spec | 8024. | 546.61 | {73., 646.} | 96.7516 | 1.5176 | newly 3 weeks cumul | 31 244. | 5527.93 | {934., 9189.} | 92.0603 | 1.1016 | newly 5 weeks spec | 4154. | 159.07 | {4., 249.} | 98.1485 | 2.09176 | newly 5 weeks cumul | 41 376. | 5973.81 | {1004., 9346.} | 92.5575 | 1.1808 | newly 53 weeks cumul | 50 863. | 6181.22 | {1020., 9350.} | 92.7913 | 1.24884 |
| 13 | newly 3 weeks spec | 7496. | 1148.32 | {58., 5337.} | 86.9453 | 1.42514 | newly 3 weeks cumul | 24 982. | 4402.4 | {457., 14 270.} | 83.1613 | 1.1445 | newly 5 weeks spec | 4998. | 1073.88 | {14., 7167.} | 95.8103 | 1.82102 | newly 5 weeks cumul | 36 270. | 6613.86 | {516., 30 476.} | 82.5811 | 1.21803 | newly 51 weeks cumul | 54 152. | 10 520.9 | {539., 59 245.} | 85.8975 | 1.34361 |
| 14 | newly 3 weeks spec | 5024. | 3196.33 | {396., 12 246.} | 94.4361 | 0.705533 | newly 3 weeks cumul | 30 056. | 23 411.3 | {3039., 86 067.} | 88.4942 | 0.565475 | newly 5 weeks spec | 1153. | 782.06 | {41., 1233.} | 111.797 | 0.86226 | newly 5 weeks cumul | 33 840. | 25 717.5 | {3926., 100 726.} | 89.6571 | 0.576787 | newly 56 weeks cumul | 34 818. | 26 808.3 | {4453., 107 883.} | 90.7573 | 0.570029 |
| 15 | newly 3 weeks spec | 5483. | 4295.12 | {525., 16 117.} | 96.8364 | 0.660547 | newly 3 weeks cumul | 26 035. | 19 243.3 | {2336., 69 512.} | 95.2128 | 0.651763 | newly 5 weeks spec | 1932. | 2172.23 | {77., 5545.} | 110.328 | 0.633697 | newly 5 weeks cumul | 31 301. | 24 411. | {3092., 96 070.} | 95.1993 | 0.62529 | newly 56 weeks cumul | 33 894. | 31 720.5 | {3786., 105 452.} | 95.1761 | 0.546238 |
| 16 | newly 3 weeks spec | 6616. | 3869.71 | {106., 13 541.} | 94.4497 | 0.928511 | newly 3 weeks cumul | 30 832. | 19 162.9 | {1797., 76 557.} | 96.5894 | 0.804966 | newly 5 weeks spec | 2584. | 1856.93 | {7., 4625.} | 107.748 | 1.11242 | newly 5 weeks cumul | 37 486. | 23 610.8 | {1814., 89 766.} | 96.1721 | 0.810741 | newly 56 weeks cumul | 41 249. | 29 266. | {1837., 105 244.} | 99.0658 | 0.78937 |
| 17 | newly 3 weeks spec | 6952. | 1137.99 | {24., 3702.} | 89.3258 | 1.34609 | newly 3 weeks cumul | 24 739. | 4997.15 | {189., 15 625.} | 85.852 | 1.22522 | newly 5 weeks spec | 4470. | 684.53 | {14., 613.} | 90.6709 | 1.5097 | newly 5 weeks cumul | 34 818. | 6566.57 | {228., 23 053.} | 85.7068 | 1.24591 | newly 51 weeks cumul | 48 456. | 8821.22 | {261., 28 876.} | 87.0653 | 1.23814 |
| 18 | newly 3 weeks spec | 7355. | 500.25 | {7., 695.} | 97.9651 | 2.02895 | newly 3 weeks cumul | 26 994. | 2962.27 | {143., 3754.} | 94.5628 | 1.44268 | newly 5 weeks spec | 4020. | 202.98 | {0., 277.} | 98.0294 | 2.74074 | newly 5 weeks cumul | 36 463. | 3489.05 | {147., 4436.} | 95.4871 | 1.52178 | newly 52 weeks cumul | 45 100. | 3861.14 | {169., 4840.} | 95.8277 | 1.57878 |
| 19 | newly 3 weeks spec | 8310. | 524. | {0., 893.} | 94.4084 | 2.08765 | newly 3 weeks cumul | 31 919. | 7856.93 | {3214., 13 887.} | 80.5889 | 0.791627 | newly 5 weeks spec | 4512. | 99.42 | {0., 233.} | 97.7965 | 2.48755 | newly 5 weeks cumul | 42 585. | 8181.85 | {3356., 14 228.} | 82.3768 | 0.880173 | newly 54 weeks cumul | 52 753. | 8283.63 | {3394., 14 282.} | 84.8207 | 0.961747 |
| 20 | newly 3 weeks spec | 6547. | 962.17 | {11., 1357.} | 88.6698 | 1.61131 | newly 3 weeks cumul | 38 691. | 7581.58 | {382., 10 836.} | 90.8844 | 1.32136 | newly 5 weeks spec | 1542. | 157.69 | {0., 549.} | 89.7737 | 1.92964 | newly 5 weeks cumul | 43 499. | 8090.97 | {386., 10 848.} | 89.8682 | 1.32995 | newly 59 weeks cumul | 44 967. | 8323.85 | {387., 10 848.} | 89.2244 | 1.32409 |
| 21 | newly 3 weeks spec | 7119. | 2669.93 | {162., 11 637.} | 88.9753 | 1.00196 | newly 3 weeks cumul | 29 356. | 18 284.9 | {1719., 82 101.} | 102.567 | 0.806497 | newly 5 weeks spec | 3172. | 897.28 | {13., 2293.} | 91.5063 | 1.31444 | newly 5 weeks cumul | 37 313. | 20 638.4 | {1848., 95 637.} | 97.0285 | 0.827954 | newly 56 weeks cumul | 42 719. | 22 824.8 | {2245., 101 759.} | 94.4616 | 0.823469 |
| 22 | newly 3 weeks spec | 6759. | 4210.63 | {558., 13 453.} | 92.8663 | 0.736226 | newly 3 weeks cumul | 29 716. | 16 957.7 | {2465., 51 145.} | 88.1595 | 0.71499 | newly 5 weeks spec | 2827. | 1758.6 | {173., 6216.} | 95.8925 | 0.749385 | newly 5 weeks cumul | 37 047. | 21 457.9 | {3209., 76 211.} | 86.6121 | 0.705636 | newly 57 weeks cumul | 41 620. | 25 314.1 | {3684., 84 551.} | 85.1254 | 0.675152 |
| 23 | newly 3 weeks spec | 6270. | 3941.63 | {58., 14 145.} | 114.155 | 1.12017 | newly 3 weeks cumul | 22 584. | 12 041.9 | {224., 58 003.} | 100.774 | 0.992464 | newly 5 weeks spec | 3912. | 1286.68 | {11., 4353.} | 82.3282 | 1.21877 | newly 5 weeks cumul | 31 596. | 15 455.1 | {295., 70 470.} | 95.1016 | 0.993207 | newly 50 weeks cumul | 43 028. | 20 175.4 | {388., 80 351.} | 89.3038 | 0.993386 |
| 24 | newly 3 weeks spec | 7567. | 2645.79 | {22., 5688.} | 81.2643 | 1.15097 | newly 3 weeks cumul | 27 322. | 9294.32 | {718., 21 786.} | 84.6475 | 0.948298 | newly 5 weeks spec | 4888. | 1924.98 | {0., 4951.} | 69.2426 | 1.44747 | newly 5 weeks cumul | 38 440. | 13 442.4 | {722., 32 904.} | 80.158 | 0.951717 | newly 50 weeks cumul | 52 801. | 26 806. | {751., 77 797.} | 74.7151 | 0.9169 |
| 25 | newly 3 weeks spec | 6782. | 649.41 | {37., 628.} | 93.73 | 1.4134 | newly 3 weeks cumul | 30 207. | 5594.71 | {1964., 7262.} | 88.7967 | 0.970992 | newly 5 weeks spec | 2743. | 343.13 | {0., 188.} | 100.783 | 2.05466 | newly 5 weeks cumul | 37 399. | 6378.15 | {2124., 7468.} | 89.559 | 1.02488 | newly 53 weeks cumul | 41 988. | 7253.26 | {2182., 7468.} | 90.7814 | 1.06052 |
| 26 | newly 3 weeks spec | 7069. | 959.08 | {38., 2948.} | 90.8895 | 1.48764 | newly 3 weeks cumul | 23 874. | 4451.23 | {252., 19 445.} | 83.2802 | 1.27639 | newly 5 weeks spec | 4701. | 805.93 | {7., 460.} | 101.756 | 1.75835 | newly 5 weeks cumul | 34 401. | 6158.6 | {289., 26 006.} | 85.705 | 1.3346 | newly 51 weeks cumul | 50 936. | 8166.91 | {309., 26 236.} | 88.5201 | 1.39239 |
| 27 | newly 3 weeks spec | 6335. | 746.44 | {62., 1223.} | 94.3728 | 1.46349 | newly 3 weeks cumul | 22 631. | 4634.78 | {504., 7105.} | 93.7203 | 1.18456 | newly 5 weeks spec | 3937. | 231.66 | {6., 468.} | 95.844 | 1.88716 | newly 5 weeks cumul | 31 837. | 5279.11 | {552., 8167.} | 93.3806 | 1.2705 | newly 52 weeks cumul | 45 079. | 5699.14 | {574., 8942.} | 92.9825 | 1.38149 |
| 28 | newly 3 weeks spec | 7243. | 3177.51 | {96., 15 075.} | 87.5804 | 0.969758 | newly 3 weeks cumul | 27 640. | 24 234.4 | {1099., 83 417.} | 95.1054 | 0.682917 | newly 5 weeks spec | 4186. | 746.14 | {15., 2173.} | 89.9575 | 1.51302 | newly 5 weeks cumul | 37 414. | 26 471.7 | {1160., 97 962.} | 89.1046 | 0.744516 | newly 56 weeks cumul | 48 001. | 27 397.9 | {1160., 102 472.} | 83.9809 | 0.803131 |
| 29 | newly 3 weeks spec | 6769. | 3818.59 | {91., 17 811.} | 103.339 | 1.15445 | newly 3 weeks cumul | 23 489. | 16 633.8 | {396., 73 312.} | 113.166 | 1.03758 | newly 5 weeks spec | 4398. | 888.15 | {36., 2990.} | 83.4986 | 1.29443 | newly 5 weeks cumul | 33 395. | 19 337.1 | {530., 82 615.} | 101.599 | 1.06378 | newly 50 weeks cumul | 46 315. | 21 354.8 | {666., 86 320.} | 91.4861 | 1.08605 |
| 30 | newly 3 weeks spec | 8192. | 1143.83 | {7., 358.} | 99.9449 | 1.92948 | newly 3 weeks cumul | 34 290. | 5056.16 | {333., 2106.} | 98.2915 | 1.46028 | newly 5 weeks spec | 3775. | 429.99 | {0., 151.} | 98.066 | 2.52273 | newly 5 weeks cumul | 43 617. | 6229.33 | {333., 2230.} | 97.8449 | 1.5227 | newly 59 weeks cumul | 50 418. | 6882.09 | {333., 2423.} | 96.8789 | 1.56177 |
| 31 | newly 3 weeks spec | 7625. | 789.77 | {28., 941.} | 95.6796 | 1.82576 | newly 3 weeks cumul | 37 142. | 5471.01 | {267., 8199.} | 92.4281 | 1.33233 | newly 5 weeks spec | 2494. | 252.52 | {2., 89.} | 99.2173 | 2.26095 | newly 5 weeks cumul | 43 977. | 6167.57 | {306., 10 183.} | 92.5374 | 1.3784 | newly 60 weeks cumul | 46 875. | 6516.44 | {308., 11 392.} | 92.4934 | 1.39758 |
| 32 | newly 3 weeks spec | 6240. | 2603.34 | {298., 4603.} | 89.7397 | 0.825473 | newly 3 weeks cumul | 27 473. | 11 445.6 | {1555., 18 334.} | 89.587 | 0.791009 | newly 5 weeks spec | 2353. | 1197.11 | {114., 1716.} | 90.371 | 0.78108 | newly 5 weeks cumul | 33 807. | 14 354.9 | {1911., 23 368.} | 88.4827 | 0.779983 | newly 57 weeks cumul | 37 286. | 18 118.8 | {2049., 73 473.} | 88.3144 | 0.73591 |
| 33 | newly 3 weeks spec | 8467. | 686.1 | {0., 1821.} | 92.0548 | 2.28003 | newly 3 weeks cumul | 28 949. | 13 124.7 | {543., 19 068.} | 83.3167 | 0.809631 | newly 5 weeks spec | 5209. | 286.65 | {0., 1188.} | 94.6218 | 3.15617 | newly 5 weeks cumul | 40 750. | 13 802.4 | {553., 19 761.} | 82.6205 | 0.899077 | newly 55 weeks cumul | 56 254. | 14 976. | {554., 25 106.} | 82.0636 | 0.975812 |
| 34 | newly 3 weeks spec | 6053. | 1547.18 | {0., 5850.} | 86.9995 | 1.90272 | newly 3 weeks cumul | 19 976. | 7130.58 | {1282., 28 619.} | 84.963 | 0.835941 | newly 5 weeks spec | 4286. | 712.31 | {0., 1398.} | 89.3077 | 2.22157 | newly 5 weeks cumul | 29 425. | 8784.14 | {1582., 37 820.} | 81.5097 | 0.871492 | newly 50 weeks cumul | 43 351. | 17 694.7 | {1958., 57 141.} | 75.7787 | 0.821071 |
| 35 | newly 3 weeks spec | 8823. | 430.99 | {0., 1144.} | 95.1152 | 2.39049 | newly 3 weeks cumul | 31 843. | 9481.51 | {407., 12 953.} | 87.5589 | 1.00562 | newly 5 weeks spec | 5272. | 189.88 | {0., 557.} | 96.3983 | 3.48619 | newly 5 weeks cumul | 44 053. | 9938.81 | {407., 13 176.} | 86.6388 | 1.09961 | newly 55 weeks cumul | 57 367. | 10 550.2 | {467., 14 237.} | 86.1172 | 1.16973 |
| 36 | newly 3 weeks spec | 6496. | 861.51 | {101., 913.} | 95.7597 | 1.34735 | newly 3 weeks cumul | 23 327. | 6208.67 | {994., 10 898.} | 89.8888 | 0.94312 | newly 5 weeks spec | 4189. | 273.16 | {6., 279.} | 97.8658 | 2.00199 | newly 5 weeks cumul | 32 804. | 6964.77 | {1096., 11 262.} | 91.1609 | 1.04389 | newly 53 weeks cumul | 44 915. | 7330.97 | {1119., 11 305.} | 91.6139 | 1.15323 |
| 37 | newly 3 weeks spec | 6929. | 3632.94 | {131., 8457.} | 100.676 | 0.956349 | newly 3 weeks cumul | 31 730. | 18 773.9 | {4760., 69 870.} | 93.7245 | 0.670458 | newly 5 weeks spec | 2540. | 1391.5 | {2., 3461.} | 106.743 | 1.27624 | newly 5 weeks cumul | 38 553. | 22 442.5 | {5469., 89 784.} | 94.3927 | 0.688417 | newly 58 weeks cumul | 42 395. | 24 534.1 | {5824., 90 349.} | 93.6697 | 0.68702 |
| 38 | newly 3 weeks spec | 5419. | 3421.6 | {389., 3306.} | 87.1297 | 0.586916 | newly 3 weeks cumul | 32 641. | 19 395.5 | {7621., 30 465.} | 77.2176 | 0.481979 | newly 5 weeks spec | 1229. | 1119.36 | {22., 1482.} | 84.1448 | 0.654582 | newly 5 weeks cumul | 36 650. | 22 428.2 | {7967., 31 298.} | 77.6908 | 0.474095 | newly 58 weeks cumul | 37 768. | 24 207.4 | {8436., 31 378.} | 76.4959 | 0.452532 |
| 39 | newly 3 weeks spec | 5970. | 909.53 | {0., 2689.} | 96.5476 | 2.00424 | newly 3 weeks cumul | 21 972. | 4418.66 | {183., 11 488.} | 94.8033 | 1.35653 | newly 5 weeks spec | 3152. | 382.45 | {0., 549.} | 97.4438 | 2.32504 | newly 5 weeks cumul | 29 579. | 5399.21 | {196., 14 808.} | 94.9795 | 1.42332 | newly 52 weeks cumul | 36 448. | 6069.89 | {196., 16 888.} | 94.9901 | 1.47426 |
| 40 | newly 3 weeks spec | 6214. | 475.82 | {65., 1146.} | 92.3428 | 1.39088 | newly 3 weeks cumul | 22 734. | 5568.7 | {815., 10 894.} | 88.2889 | 1.00011 | newly 5 weeks spec | 3630. | 105.84 | {3., 200.} | 97.0843 | 1.98069 | newly 5 weeks cumul | 31 216. | 5884.68 | {884., 11 270.} | 89.0144 | 1.0898 | newly 53 weeks cumul | 41 146. | 6027.87 | {913., 12 087.} | 89.885 | 1.18625 |
| 41 | newly 3 weeks spec | 5376. | 1838.04 | {220., 4804.} | 72.753 | 0.819271 | newly 3 weeks cumul | 21 206. | 14 328.4 | {1924., 38 686.} | 92.2337 | 0.633764 | newly 5 weeks spec | 2830. | 548.28 | {36., 1776.} | 84.6693 | 1.25343 | newly 5 weeks cumul | 27 896. | 15 823.9 | {2016., 40 588.} | 84.6731 | 0.675554 | newly 54 weeks cumul | 33 607. | 16 805.5 | {2046., 40 773.} | 79.7343 | 0.711333 |
| 42 | newly 3 weeks spec | 4668. | 2425.22 | {219., 2334.} | 95.0488 | 0.746862 | newly 3 weeks cumul | 24 350. | 11 267.8 | {3054., 16 253.} | 86.7164 | 0.660359 | newly 5 weeks spec | 1451. | 984.88 | {34., 1241.} | 96.2495 | 0.792702 | newly 5 weeks cumul | 28 470. | 13 802.7 | {3565., 16 688.} | 88.109 | 0.658548 | newly 58 weeks cumul | 30 111. | 15 527. | {3852., 16 718.} | 87.8177 | 0.63388 |
| 43 | newly 3 weeks spec | 8069. | 1752.94 | {118., 4912.} | 83.7799 | 1.11907 | newly 3 weeks cumul | 29 202. | 7410.73 | {1014., 11 721.} | 88.0419 | 0.988138 | newly 5 weeks spec | 4750. | 1629.73 | {40., 7055.} | 89.3754 | 1.34069 | newly 5 weeks cumul | 40 208. | 10 707.9 | {1192., 26 010.} | 82.3783 | 0.990967 | newly 51 weeks cumul | 53 458. | 19 257.3 | {1339., 68 474.} | 80.483 | 1.01996 |
| 44 | newly 3 weeks spec | 6354. | 3826.8 | {801., 12 964.} | 78.2128 | 0.546439 | newly 3 weeks cumul | 27 932. | 17 133.7 | {3199., 34 043.} | 81.5272 | 0.555864 | newly 5 weeks spec | 2650. | 1898.67 | {338., 2410.} | 83.8721 | 0.521314 | newly 5 weeks cumul | 34 711. | 21 672.1 | {4251., 61 557.} | 79.5571 | 0.539903 | newly 57 weeks cumul | 39 102. | 26 979.5 | {5367., 98 417.} | 78.3913 | 0.49726 |
| 45 | newly 3 weeks spec | 4797. | 2475.38 | {296., 9658.} | 88.0179 | 0.760333 | newly 3 weeks cumul | 26 104. | 17 836.6 | {1970., 74 035.} | 101.749 | 0.701802 | newly 5 weeks spec | 1420. | 897.81 | {32., 1362.} | 89.5528 | 0.823075 | newly 5 weeks cumul | 30 202. | 20 086.5 | {2418., 88 496.} | 97.9037 | 0.685596 | newly 56 weeks cumul | 31 840. | 23 291.1 | {2735., 94 146.} | 97.5975 | 0.646514 |
| 46 | newly 3 weeks spec | 8356. | 2279.09 | {82., 7905.} | 84.3457 | 1.02896 | newly 3 weeks cumul | 33 903. | 18 415. | {1771., 34 558.} | 93.4306 | 0.769791 | newly 5 weeks spec | 3539. | 640.05 | {3., 942.} | 91.7494 | 1.43824 | newly 5 weeks cumul | 42 756. | 20 179.5 | {2160., 56 947.} | 90.5364 | 0.80274 | newly 56 weeks cumul | 48 435. | 21 460.3 | {2256., 87 792.} | 89.2309 | 0.807516 |
| 47 | newly 3 weeks spec | 6682. | 3259.43 | {392., 8251.} | 97.383 | 0.836958 | newly 3 weeks cumul | 30 081. | 19 476.6 | {3123., 68 862.} | 98.2957 | 0.66971 | newly 5 weeks spec | 2681. | 1004.24 | {31., 2572.} | 88.9467 | 1.0576 | newly 5 weeks cumul | 37 114. | 22 244.4 | {3859., 95 945.} | 96.1918 | 0.685157 | newly 58 weeks cumul | 40 818. | 23 822.3 | {4243., 96 109.} | 94.136 | 0.676392 |
| 48 | newly 3 weeks spec | 8702. | 3680.85 | {235., 18 391.} | 90.4864 | 0.871459 | newly 3 weeks cumul | 37 680. | 18 288.6 | {5323., 70 664.} | 85.8287 | 0.655292 | newly 5 weeks spec | 3585. | 1123.58 | {10., 2657.} | 84.6996 | 1.10716 | newly 5 weeks cumul | 46 811. | 21 445.5 | {6058., 87 039.} | 85.2809 | 0.676788 | newly 58 weeks cumul | 52 174. | 23 076.9 | {6463., 91 001.} | 83.8281 | 0.676558 |
| 49 | newly 3 weeks spec | 4127. | 313.94 | {35., 389.} | 96.6654 | 1.62759 | newly 3 weeks cumul | 27 465. | 3030.09 | {524., 3785.} | 89.7807 | 1.28273 | newly 5 weeks spec | 921. | 111.48 | {2., 33.} | 104.332 | 2.03488 | newly 5 weeks cumul | 30 359. | 3320.82 | {556., 4124.} | 90.6773 | 1.30894 | newly 60 weeks cumul | 31 206. | 3501.25 | {557., 4176.} | 90.9231 | 1.31721 |
| 50 | newly 3 weeks spec | 6476. | 1535.59 | {103., 2783.} | 87.4294 | 1.13521 | newly 3 weeks cumul | 23 838. | 5848.79 | {1166., 19 155.} | 77.749 | 0.891586 | newly 5 weeks spec | 3992. | 1036.93 | {7., 2092.} | 95.9502 | 1.48416 | newly 5 weeks cumul | 32 964. | 8198.28 | {1316., 20 668.} | 81.6098 | 0.94741 | newly 51 weeks cumul | 44 879. | 11 606.9 | {1342., 34 319.} | 80.6528 | 0.980056 |
